# Molecular signature of postmortem lung tissue from COVID-19 patients suggests distinct trajectories driving mortality

**DOI:** 10.1242/dmm.049572

**Published:** 2022-06-06

**Authors:** Anshul Budhraja, Anubhav Basu, Atish Gheware, Dasari Abhilash, Seesandra Rajagopala, Suman Pakala, Madhuresh Sumit, Animesh Ray, Arulselvi Subramaniam, Purva Mathur, Aruna Nambirajan, Sachin Kumar, Ritu Gupta, Naveet Wig, Anjan Trikha, Randeep Guleria, Chitra Sarkar, Ishaan Gupta, Deepali Jain

**Affiliations:** 1 Department of Biochemical Engineering and Biotechnology, Indian Institute of Technology, New Delhi 110016, India; 2 Department of Pathology, All India Institute of Medical Sciences, New Delhi 110029, India; 3 Department of Medicine, Division of Infectious Diseases, Vanderbilt University Medical Center, Nashville, TN 37232, USA; 4 Department of Medicine, All India Institute of Medical Sciences, New Delhi, 110029, India; 5 Department of Laboratory Medicine, JPNATC, All India Institute of Medical Sciences, New Delhi 110029, India; 6 Department of Medical Oncology, All India Institute of Medical Sciences, New Delhi 110029, India; 7 Laboratory Oncology, Dr. B. R. Ambedkar Institute Rotary Cancer Hospital (IRCH), All India Institute of Medical Sciences, New Delhi 110029, India; 8 Department of Anaesthesiology, Critical Care and Pain Medicine, All India Institute of Medical Sciences, New Delhi 110029, India; 9 Department of Pulmonary Medicine and Sleep Disorders, All India Institute of Medical Sciences, New Delhi 110029, India

**Keywords:** SARS-CoV-2, Infectious disease, Metatranscriptomics, Whole-transcriptome sequencing, Lung microbiome

## Abstract

To elucidate the molecular mechanisms that manifest lung abnormalities during severe acute respiratory syndrome coronavirus 2 (SARS-CoV-2) infections, we performed whole-transcriptome sequencing of lung autopsies from 31 patients with severe COVID-19 and ten uninfected controls. Using metatranscriptomics, we identified the existence of two distinct molecular signatures of lethal COVID-19. The dominant ‘classical’ signature (*n*=23) showed upregulation of the unfolded protein response, steroid biosynthesis and complement activation, supported by massive metabolic reprogramming leading to characteristic lung damage. The rarer signature (*n*=8) that potentially represents ‘cytokine release syndrome’ (CRS) showed upregulation of cytokines such as IL1 and CCL19, but absence of complement activation. We found that a majority of patients cleared SARS-CoV-2 infection, but they suffered from acute dysbiosis with characteristic enrichment of opportunistic pathogens such as *Staphylococcus cohnii* in ‘classical’ patients and *Pasteurella multocida* in CRS patients. Our results suggest two distinct models of lung pathology in severe COVID-19 patients, which can be identified through complement activation, presence of specific cytokines and characteristic microbiome. These findings can be used to design personalized therapy using *in silico* identified drug molecules or in mitigating specific secondary infections.

## Abstract (precis)

**Editor's choice:** This is the first study analyzing the lung transcriptome in the Indian population, revealing that COVID-19 patients with complications who die of severe symptoms might have at least two distinct molecular trajectories.

## INTRODUCTION

Despite numerous interventions, the novel severe acute respiratory syndrome coronavirus 2 (SARS-CoV-2) continues to cause significant morbidity and mortality throughout the world. As of May 2022, India alone had diagnosed over 43.1 million people with this virus, with over 524,000 fatalities (Dong et al., 2020). Although the disease caused by SARS-CoV-2 infection, COVID-19, is believed to progress often asymptomatically or with only mild to moderate symptoms, primarily fever and dry cough, in many instances it can exacerbate acute pneumonia, especially in susceptible patients such as older individuals with metabolic, cardiovascular and/or pulmonary comorbidities (Mehrian-Shai, 2020; Tay et al., 2020).

As has been reported, SARS-CoV-2 enters the host cell using the angiotensin-converting enzyme 2 (ACE2) receptor, which binds to the viral spike (S) protein's receptor-binding domain (RBD) (Hoffmann et al., 2020). The viral genome is released into the cytoplasm once the viral envelope fuses with the host cell membrane in a Toll-like receptor 7 (TLR-7)-dependent manner (Ahmadpoor and Rostaing, 2020). The virus uses its own RNA-dependent RNA polymerase to replicate its genome (Simmons et al., 2013; Sexton et al., 2016). The replication-transcription complex (RTC) is formed in a double-membrane vesicle (Sawicki and Sawicki, 2005) by two large polyproteins (pp1a and pp1b), which encode non-structural proteins (Millet and Whittaker, 2015). The continuous replication by the RTC results in the formation of many sub-genomic RNAs (Hussain et al., 2005) that code for structural and auxiliary proteins. Virus assembly and budding takes place in smooth-walled vesicles in the endoplasmic reticulum–Golgi intermediate compartment (ERGIC) (Masters, 2006), and, finally, the virion-containing vesicles fuse with the plasma membrane to release the virus by exocytosis.

Many studies have established a strong link between the regulation of the innate immune response, the development of adaptive immunity and the severity of COVID-19 (Mason, 2020; Mehrian-Shai, 2020; Tay et al., 2020). A hyperinflammatory response was found in patients' blood, nasopharyngeal samples and bronchoalveolar lavage fluid (BALF), as evidenced by increased levels of cytokines such as IL6, TNF-α and MCP-1, which can lead to severe acute respiratory syndrome (SARS), extensive coagulopathy and multiorgan failure (Mason, 2020; Mehrian-Shai, 2020; Tay et al., 2020). Therefore, patients with severe COVID-19 require oxygen supplementation and intensive care, potentially exposing them to secondary opportunistic infections (Clancy and Nguyen, 2020; Friedland and Haribabu, 2020; Rawson et al., 2020; Ripa et al., 2021). As a result, current guidelines suggest the use of anticoagulant, anti-inflammatory and antiviral medication along with broad-spectrum antibiotics and antifungals in patients with suspected or confirmed COVID-19 (Clancy and Nguyen, 2020; Friedland and Haribabu, 2020; Mehrian-Shai, 2020; Rawson et al., 2020). However, even with the same clinical intervention, patients display distinct trajectories with vastly different recovery times, clinical outcomes or mortality (Jayaswal et al., 2021). The molecular origin of such diverse outcomes are poorly understood in the context of lung pathophysiology, and only a handful of primary datasets with smaller sample sizes have been published (Wu et al., 2020b; Nienhold et al., 2020; Sanchez-Cerrillo et al., 2020; Xiong et al., 2020; Zhou et al., 2020).

This situation is further complicated by the emergence of coinfections in COVID-19 patients due to immunosuppression that can cause mucormycosis (Gupta et al., 2021; Moona and Islam, 2021; Prakash and Chakrabarti, 2021; Sen et al., 2021). Studies during previous SARS and Middle East respiratory syndrome (MERS) epidemics showed that individuals receiving invasive mechanical ventilation were more likely to develop secondary infections and have a higher fatality rate (Feldman and Anderson, 2021). Recent investigations indicate that coinfections and/or superinfections occur at varying frequencies in oral, blood and urine samples from COVID-19 patients (Charalampous et al., 2020; Langford et al., 2020; Mostafa et al., 2020; Alhumaid et al., 2021; Ripa et al., 2021; Rodriguez et al., 2021; Silva et al., 2021; Vijay et al., 2021). However, little is known about the prevalence of these pathogens and their exact molecular relevance in the human lung tissue during COVID-19 infection. Given that most COVID-19 deaths are due to pneumonia-related complications, it is critical to identify pathogens that co-infect severe COVID-19 patients and perform targeted therapeutic interventions.

In this work, we performed whole-transcriptome sequencing of autopsy lung tissue from 31 patients who died due to severe COVID-19 related complications, and compared them to lung biopsies from ten control patients who were not infected with SARS-CoV-2. Using metatranscriptomics, we determined characteristic changes to the host transcriptome and unique microbial diversity in the lung parenchyma of severe COVID-19 patients. We mapped the host response at the level of genes, pathways and change in cell-type abundances, and also identified unique microbiome signatures driving dysbiosis in severe COVID-19 patients. Further, we correlate these findings with clinical features of the disease and dissect the potential molecular etiology of the disease that might help explain diverse outcomes leading to complications and suggest potential personalized therapeutic interventions.

## RESULTS

**Fig. 1.**
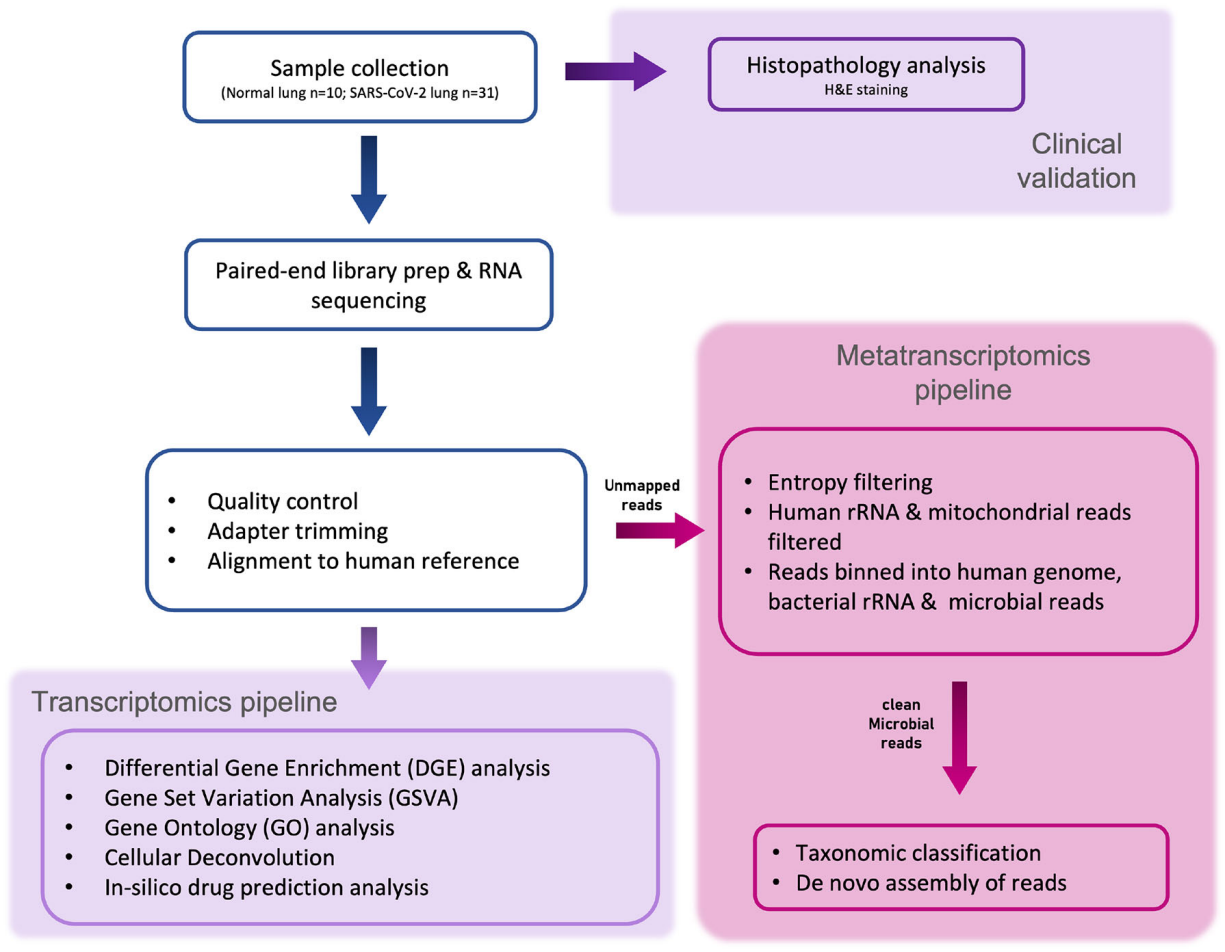
**Schematic for histopathological and metatranscriptomic analysis pipeline.** Lung tissue biopsy samples were collected from postmortem COVID-19-affected and control patients. The tissue samples were first evaluated for clinical physiology. The host rRNA was depleted prior to NGS library preparation, and the samples were then sequenced at an average depth of ∼47 million reads per sample. The sequence reads were filtered based on read quality. Filtered reads were used for differential gene expression, virome and microbiome profiling.

To characterize the pathology of SARS-CoV-2 infection, postmortem lung tissue samples were collected from 31 patients who had been diagnosed with severe COVID-19. As a control, ten uninfected normal lung samples were taken from patients diagnosed with cancer as part of standard surgery procedures (Fig. 1). Among the 41 samples, 19 (46%) were from females and 22 (54%) from males. Out of the ten control samples, six were from females and four from males, whereas in the 31 COVID-19 samples, 13 were from females and 18 from males. The mean age for all 41 patients was 51.65±15.27 years. The mean age for patients from the control group was 51±16.22 years, whereas that of the COVID-19 patients was 57±15.23 years (Table 1). Using nasopharyngeal swab PCR, all cases tested positive for SARS-CoV-2. At the time of onset, the most common symptoms were shortness of breath, fever and cough.

**Table 1.**
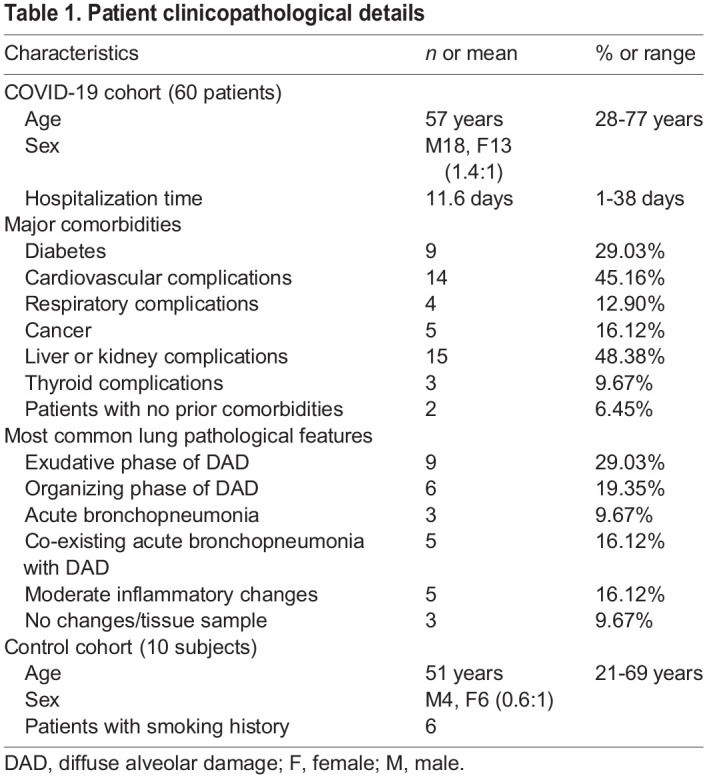
Patient clinicopathological details

**Fig. 2.**
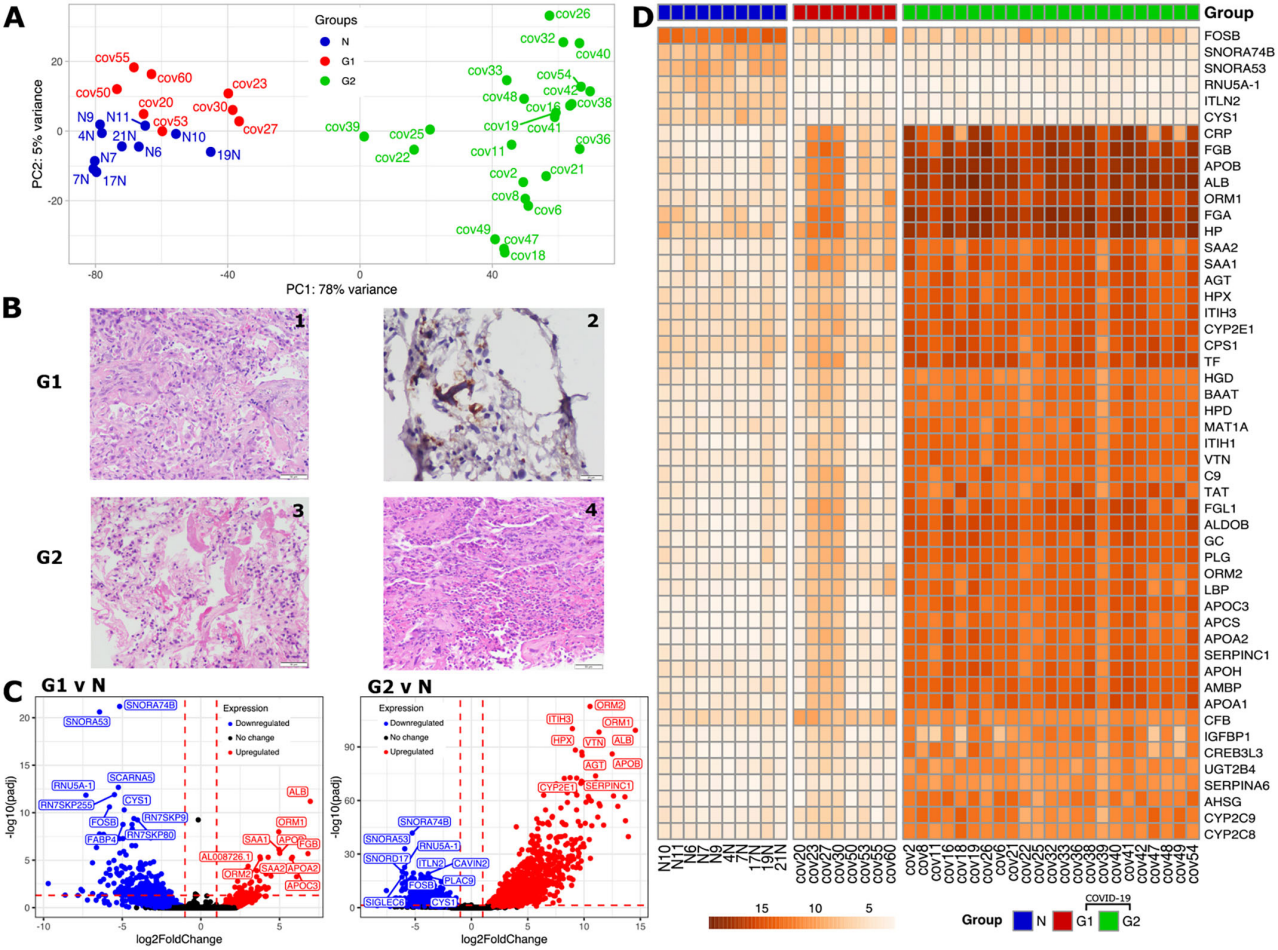
**Differential expression and histopathological analyses of COVID-19 samples.** (A) PCA plot based on gene expression counts across COVID-19 samples and the control group (‘N’, blue) identified two groups of patients, G1 (red) and G2 (green). (B) Representative histological images of postmortem lung tissue sections for the two patient groups. The G1 group (above) displayed acute organizing pneumonia (200× magnification) (1) and microthrombi (CD61 immunostaining, 400× magnification) (2). The G2 group (below), displayed diffuse alveolar damage with hyaline membranes (200× magnification) (3) and acute bronchopneumonia with microabscesses (200× magnification) (4). Scale bars: 50 μm (panels 1,3,4), 20 μm (panel 2). (C) Volcano plot describing the fold changes and false discovery rate (FDR)-adjusted *P*-values between the two groups of COVID-19 patients versus control samples with downregulated genes in blue and upregulated genes in red. The top ten differentially expressed (DE) gene names are highlighted. The horizontal dashed line indicates the significance cut-off [adjusted *P*-value (*P*_adj_)<0.05; Wald test, corrected for multiple testing using the Benjamini–Hochberg method] and the vertical dashed lines indicate the log_2_ fold change (l2fc) cutoffs (l2fc>1 and l2fc<−1). (D) Variance-stabilizing transformed (vst) gene expression profile of the top 50 significantly DE genes between control and two groups of COVID-19 patients.

The major cause of mortality was respiratory failure or multiorgan failure affecting the respiratory system. Consistent with a previous report (Carsana et al., 2020), the pulmonary tissue of all deceased COVID-19 patients was highly abnormal, with evidence of diffuse alveolar damage (DAD), in addition to widespread hyaline membrane formation (the pathological hallmark of acute respiratory distress syndrome), acute lung injury, bronchopneumonia and thrombosis being frequent (Fig. 2B; Fig. S2G, Table S1). COVID-19-afflicted patients' lungs also showed varying degrees of an inflammatory infiltrate.

To dissect the molecular aberrations underlying fatal COVID-19 cases, 41 lung tissue samples (31 cases and ten controls) were subjected to whole-transcriptome analysis using RNA sequencing (RNA-seq). The average number of reads ranged from 13.3 million to 115 million with an average of 46.9 million reads per sample. The range for the ten control samples was 35.4 million to 115 million with a mean of 58.9 million reads, whereas the range for the 31 COVID-19 samples was slightly lower at 13.3 million to 67.2 million with an average of 43.1 million reads, potentially owing to lung damage (Fig. S1A). The RNA-seq data were aligned to the human reference genome GRCh38 (GENCODE v36) to summarize gene counts. The alignment rate ranged from 60.0% to 89.6% with a mean±s.d. of 83.72±6.72% (Fig. S1B).

### Lung transcriptome signature in severe COVID-19

To interpret the host response, differential expression (DE) analysis was performed using the DESeq2 package in R accounting for confounding variables such as age and sex. By visualizing the gene level data per sample in a principal component analysis (PCA) plot, we found that the controls and the majority of the COVID-19 samples clearly segregated on the first principal component (PC) (explaining 78% variance), except for eight COVID-19 samples (cov20, cov23, cov27, cov30, cov50, cov53, cov55 and cov60) that clustered with control or normal samples (indicated by ‘N’). Henceforth, we refer to these eight samples as G1 (group 1) and the rest of the COVID-19 samples as G2 (group 2), which can be seen colored differently in the PCA plot (Fig. 2A). Next, we tried to check whether the gene expression patterns of the G1 and G2 samples could be explained by the presence of any comorbidities. However, our analysis (Table S1) found no significant correlations (Fig. S2A-F).

We identified a total of 1856 significantly differentially expressed genes (DEGs) between control and all COVID-19 samples, with 864 genes significantly upregulated and 992 genes significantly downregulated (Materials and Methods) in COVID-19 patients (Fig. 2C). DE analysis between the G1 and control (normal or ‘N’) samples revealed the presence of 263 significant DEGs, with 56 genes significantly upregulated and 207 genes significantly downregulated, whereas, in the case of G2 group and control samples, there were 3094 DEGs, out of which 1363 were significantly upregulated and 1731 were significantly downregulated (Table S3). Between the two COVID-19 groups G2 and G1, there were 1433 DEGs, out of which 1314 were significantly upregulated and 119 were significantly downregulated (Fig. 2C). Further DE analysis of G2 and G1 can be found in Fig. S3. A heat map plotting the variance stabilized (vst) gene expression of the top 50 differentially expressed genes between all COVID-19 and normal samples clearly divides the samples into two groups and reaffirms the presence of two distinct categories of COVID-19 samples, with gene expression of G1 matching that of normal samples (Fig. 2D). We observed that 189 genes were differentially expressed in both G1 compared to N and G2 compared to N, whereas 2899 genes were only differentially expressed in G2 compared to N, and 74 genes were only differentially expressed in G1 compared to N (Table S3).

The most significantly upregulated genes in G1 and G2 include orosomucoid 1 (*ORM1*, better known as alpha-1-acid glycoprotein 1 or *AGP1*), orosomucoid 2 (*ORM2*, also known as alpha-1-acid glycoprotein 2 or *AGP2*), apolipoprotein B (*APOB*) and albumin (*ALB*) (Fig. 2C,D). Orosomucoid is known to be regulated by TNF-β, IL1β, IL6 and IL6-related cytokines (Baumann et al., 1989; Fournier et al., 2000), along with immunomodulating effects like inhibiting neutrophil migration (Mestriner et al., 2007), and has been employed as a biomarker in COVID-19 (Shu et al., 2020). APOB has been observed to be upregulated in enterovirus 71 infection (Leong and Chow, 2006) and displays elevated levels in the blood of COVID-19 patients (Pushkarev et al., 2021). ALB levels in blood can be used as a biomarker for measuring COVID-19 severity (Liang et al., 2021).

Inter-α-trypsin inhibitor heavy chain 3 (*ITIH3*), hemopexin (*HPX*), vitronectin (*VTN*) and angiotensinogen (*AGT*), *SERPINC1* and *CYP2E1* are genes that are highly upregulated in G2 when compared to normal samples (Fig. 2C,D). ITIH3 has been indicated as one of the plasma mortality markers for COVID-19 (Demichev et al., 2020 preprint; Völlmy et al., 2021). AGT is a hormone precursor involved in the blood pressure regulation cascade, and is implicated as a potential biomarker and linked to the severity of COVID-19 (Sriram and Insel, 2020; Kouhpayeh et al., 2021). VTN levels in platelets were observed to be high in the case of SARS-CoV pneumonia as well (Lazzaroni et al., 2021). It has been noted that CYP2E1 has played a role in oxidative stress in hepatitis C (Smirnova et al., 2016), and increased CYP2E1 levels have been associated with higher risk of adverse events, such as hepatotoxicity, especially in potential COVID-19 patients with obesity and metabolic (dysfunction)-associated fatty liver disease (MAFLD) as a comorbidity (Ferron et al., 2020).

Some of the top ten genes upregulated in G1 are fibrinogen β chain (*FGB*), serum amyloid A1 (*SAA1*), serum amyloid A2 (*SAA2*), apolipoprotein A2 (*APOA2*), apolipoprotein C3 (*APOC3*) and *AL008726.1* (Fig. 2C). Increased abundance of fibrinogen-β (FGB), also found in SARS-CoV-2 infected plasma exosomes, is known to stimulate pro-inflammatory cytokine signaling (Sur et al., 2021). Proteomics studies showed the signatures of cytokine production and interferon-γ response, and increased levels of SAA1 in the serum of COVID-19 patients (Singh et al., 2021). SAA2 might be a predictor of the severity of COVID-19 (Papoutsoglou et al., 2021).

The small nucleolar RNAs, H/ACA Box 74B (SNORA74B), H/ACA Box 53 (SNORA53) and RNU5A-1, along with the proteins FOSB and cystin 1 (CYS1) are significantly downregulated in both G1 and G2, when compared to control samples (Fig. 2C,D). Downregulation of CYS1 has been observed in another study for SARS-CoV-2, wherein low levels of CYS1 have been linked to activation of NF-κB and the subsequent cytokine storm (Zolfaghari Emameh et al., 2020). FOSB inactivation in mast cells has been shown to increase the inflammatory response (Duque-Wilckens et al., 2021), and it has contradictorily also been reported to be upregulated in single cell analysis of CD4^+^ T-cells of severe COVID-19 patients (Kalfaoglu et al., 2020).

*SNORD17*, *ITLN2*, *CAVIN2*, *PLAC9* and *SIGLEC6* are some of the genes highly downregulated in G2, along with *SCARNA5*, *FABP4* and pseudogenes (namely *RN7SKP255*, *RN7SKP9* and *RN7SKP80*) being highly downregulated in G1 (Fig. 2C). As seen in G2, interlectin 2 (ITLN2) was found to be downregulated in a study of 22 blood samples of severe COVID-19 patients as well (Vastrad et al., 2020). Contrary to the trend observed in G2, in a single-cell sequencing study of 16 COVID-19 patients, megakaryocyte progenitor cells/platelets showed increased expression of CAVIN2. SIGLEC6 belongs to the family of sialic acid-binding immunoglobulin-like lectin proteins, out of which SIGLEC1, SIGLEC7 and SIGLEC10 have been implicated to play a role in COVID-19 (Doehn et al., 2021; Saheb Sharif-Askari et al., 2021). Low levels of fatty acid-binding protein 4 (FABP4) in BALF macrophages of patients suffering from severe COVID-19 have been linked to declining lung function (Liao et al., 2020).

**Fig. 3.**
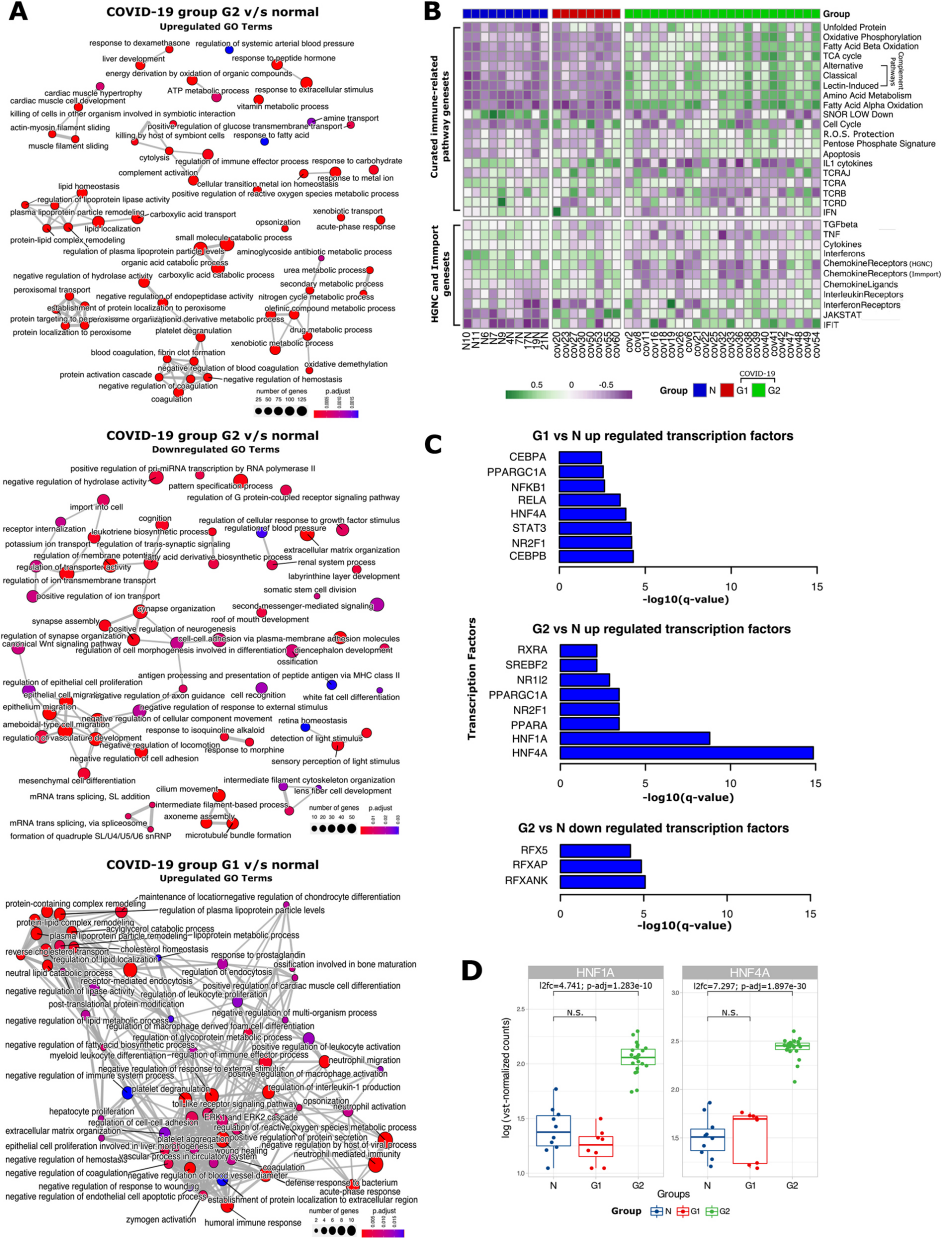
**Gene Ontology (GO) enrichment, gene set variation analysis (GSVA) and transcription factor analysis between the G1 and G2 COVID-19 groups and the control group.** (A) GO enrichment map with nodes representing biological processes, edges representing overlapping gene sets. Upregulated biological processes in G2 versus control (‘N’; top). Downregulated biological processes in G2 versus N (middle). Upregulated biological processes in G1 versus N (bottom). (B) Heatmap of GSVA depicts profiles of curated gene sets (Daamen et al., 2021; Vasiliou et al., 2021; Bhattacharya et al., 2018) across all the samples in our study. (C) Bar plots indicate the transcription factor targets enriched in significantly upregulated genes in G1 versus N, upregulated genes in G2 versus N and downregulated genes in G2 versus N. (D) Box plots of log-transformed vst-normalized gene counts representing differences among G1, G2 and normal sample groups for *HNF1A* and *HNF4A*. The box represents the 25-75th percentiles, and the median is indicated. The whiskers show the range. l2fc, log_2_ fold change; N.S., not significant. Statistical significance was determined using the Wald test, corrected for multiple testing using the Benjamini–Hochberg method; *P*_adj_<0.05.

To identify biological processes implicated in the host response to SARS-CoV-2, we performed a Gene Ontology (GO) analysis of DEGs. The top 60 enriched GO terms (adjusted *P*-value <0.05) were organized into a network of modules with edges connecting overlapping gene sets (Fig. 3A). Key modules enriched in the G1 group included lipid metabolism, negative regulation of coagulation and neutrophil-mediated immunity, as reported previously (Wu et al., 2020b; Sanchez-Cerrillo et al., 2020; Xiong et al., 2020; Zhou et al., 2020). Key modules enriched in the G2 group were related to complement activation, xenobiotic metabolism and peroxisomal protein transport, as reported previously (Knoblach et al., 2021). No enriched modules were found to be downregulated in the G1 group, whereas modules related to cilium formation, synapse formation and membrane potential were downregulated in the G2 group, suggesting the suppression of neuronal processes as reported elsewhere (Wu et al., 2020b).

### Targeted gene set enrichment analysis using gene set variation analysis

To gain a better understanding of the regulation of key pathways identified in the GO analysis, we performed gene set variation analysis (GSVA). First, we analyzed curated lists from the Human Genome Organization (HUGO) Gene Nomenclature Committee (HGNC) and ImmPort (Table S2) of immune-related pathways, interferons, chemokines, interleukins and their receptors (https://www.genenames.org/data/genegroup/#!/). The expression of genes from 11 of these gene lists was significantly altered, with the ‘Chemokine Ligands’ genes being altered only in G1, and genes from seven of the gene lists being exclusively altered in G2 (Fig. 3B). Genes corresponding to the remaining three gene lists, namely, interleukin receptors, interferon-induced transmembrane proteins (IFITs) and JAK-STAT signaling, were upregulated in both groups, indicating a core antiviral inflammatory response (Sadler and Williams, 2008; Schoggins and Rice, 2011) in both patient groups. However, specific genes within each gene set were upregulated in each patient group, suggesting distinct molecular pathways towards inflammation (Fig. S4B). The interleukin receptor genes *IL20RA* and *IL5RA* were upregulated in the G1 group, whereas *IL17RB*, *IL1RN* and *IL22RA1* were upregulated in the G2 group. The interleukin genes *IL1RN* and *IL27* were only upregulated in G2; however, *IL6* was not upregulated in either group, corroborating previous observations in lung tissue (Wu et al., 2020b), despite high levels in the blood of COVID-19 patients (Patel et al., 2021). Similarly, the entire chemokine ligand gene set itself was only upregulated in the G1 group, potentially due to high expression of chemokines such as CCL19 (Fig. S4E). Specific chemokine genes such as *CCL16* were also upregulated only in the G2 group. We found further evidence of a distinct inflammatory response within the G2 group through the exclusive downregulation of genes corresponding to interferons, chemokine receptors, tumor necrosis factor-mediated antiviral signaling and TGF-β signaling (Fig. 3B), albeit with heterogeneity in expression of individual genes, especially bone morphogenic proteins (BMPs), growth differentiation factors (GDFs) and inhibins (Fig. S4B). Further, we found significant downregulation of genes associated with T-cell receptor signaling genes in the G2 group, indicating T-cell dysfunction and potentially aberrant response.

**Fig. 4.**
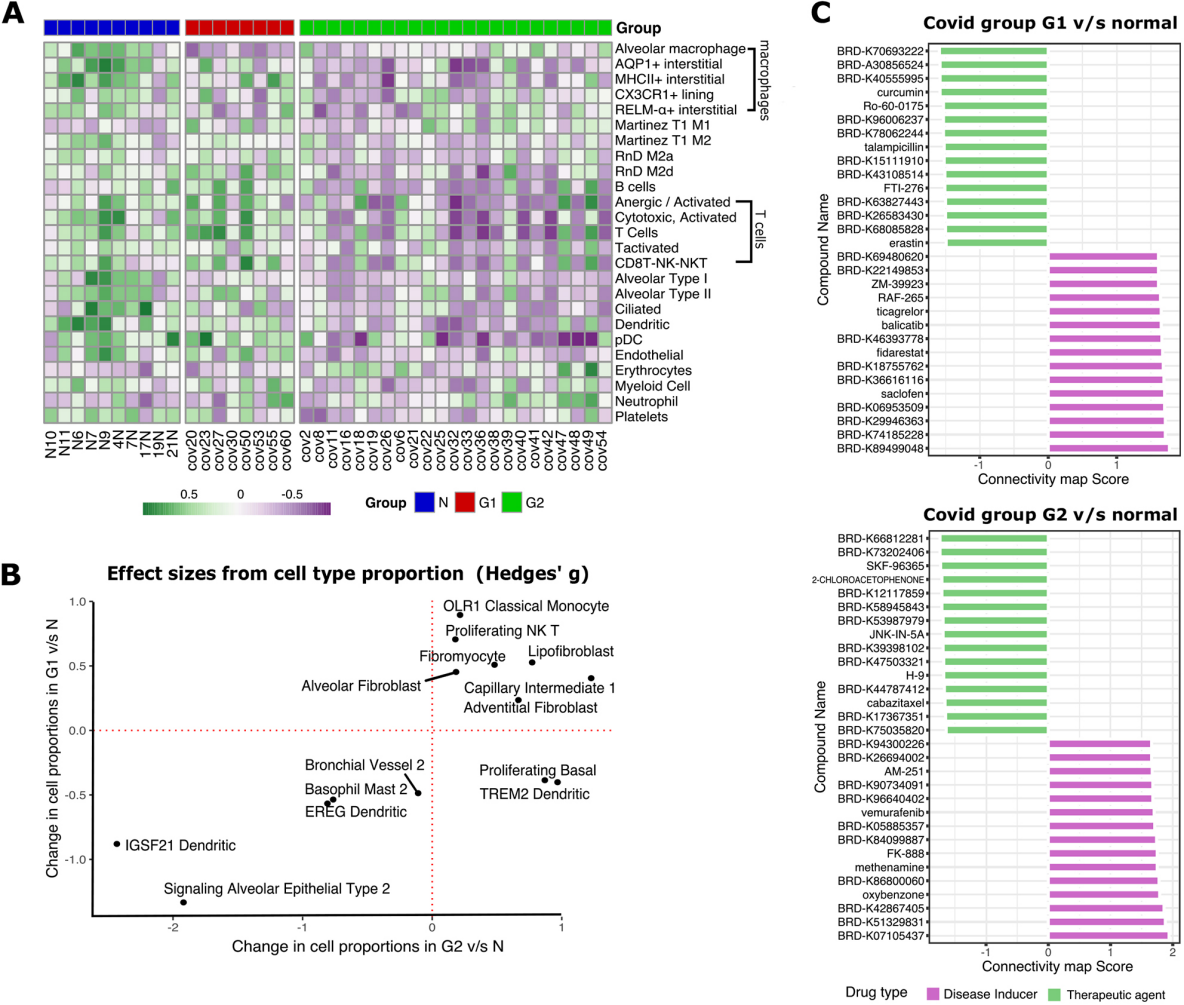
**Cell-type-specific gene set enrichment and *in silico* drug screening.** (A) Heatmap of GSVA enrichment scores per sample for previously implicated cell types involved in physical organization of lung physiology (Daamen et al., 2021) that are significantly differentially expressed (*P*<0.05, Wilcoxon test) between normal (‘N’) and either G1 or G2 samples. (B) Changes in effective size of relative cellular proportions in G1 versus N and G2 versus N. Proliferating basal cells and TREM2^+^ dendritic cells were increased in G2 patients compared to G1. (C) Bar plots of connectivity map (CMap) scores for potential disease inducers (purple) and potential therapeutic compounds (green) for G1 and G2 groups of patients, respectively.

We then dissected the molecular pathways that might be involved in the physical re-organization of lung tissue (Table S2) using GSVA. We found that genes involved in fibrosis and extracellular structure organization were significantly upregulated in both the patient groups (Fig. 4A). Corroborating previous reports in COVID-19 patients (Islam and Khan, 2020), surfactant proteins, which maintain surface tension in alveoli (Glasser and Mallampalli, 2012), were significantly downregulated. Again, individual genes within each of these gene sets displayed distinct expression patterns between the two groups, suggesting different routes towards aberrant lung physiology (Fig. S4A).

Next, we studied pathways previously found to be dysregulated in COVID-19. Out of a curated list of 36 pathways (Daamen et al., 2021), 20 (55.55%) pathways were found to be significantly upregulated between G1 or G2 groups relative to control samples. The G1 group showed significant overexpression of genes in cell cycle regulation and IL1 cytokines, whereas the G2 group showed significant overexpression of genes related to apoptosis, all three complement pathways (classical, alternative and lectin-induced) and metabolic pathways such as fatty acid oxidation, sugar metabolism, tricarboxylic acid (TCA) cycle and oxidative phosphorylation (Fig. 3B). The massive change in gene expression for metabolic pathways was further corroborated by GSVA using KEGG pathways, whereby 85 out of 186 (45.69%) pathways were significantly altered (Fig. S5A), such as upregulation of steroid biosynthesis pathways and downregulation of neuroactive ligand-receptor interaction (corresponding to symptoms of anosmia and ageusia). Both the patient groups had characteristic dysregulation of T-cell receptor (TCR) genes, confirming previous bias (Gutierrez et al., 2020; Wang et al., 2021a). For example, in G1, the GSVA scores indicated that the *TCRD* gene set [containing eight TRD genes as listed in Daamen et al. (2021)] was downregulated and the *TCRAJ* gene set (containing 56 TRAJ genes) was upregulated compared to the control group, whereas in G2, both the *TCRA* and *TCRB* gene sets (containing 45 TRAV and 45 TRB genes, respectively) were downregulated compared to the control group (Fig. 3B).

We then studied host genes that directly interact with the viral proteins to help in viral entry and infection. The expression of both *ACE2* and *TMPRSS2*, which play a role in SARS-CoV-2 entry (Hoffmann et al., 2020), did not differ between either group of patients when compared to the control group (Fig. S4D). The expression of the genes encoding cathepsins B and L, *CTSB* and *CTSL*, respectively, which can substitute for TMPRSS2 (Hoffmann et al., 2020), also remained unchanged (Fig. S4D). Next, we investigated the expression of 331 human proteins that directly bind to viral proteins (Gordon et al., 2020) in our samples (Table S2, Fig. S4C). Out of 248 (74.92%) highly expressed genes (depth greater than 100 reads in 90% of either control or COVID-19 samples, hypothesizing that high expression would indicate a direct role), we found only 16 genes, including *ERGIC1*, *GGH*, *PCSK6* and *PLOD2*, that were differentially expressed only in the G2 group, suggesting no significant over-representation of the human host interactome in our patients (*P*=0.985 using a hypergeometric test).

In a study (Nienhold et al., 2020) based on postmortem lung tissue samples from 22 patients, 398 genes were evaluated using targeted RNA-seq [Oncomine Immune Response Research Assay (OIRRA, Cat No. A32881, ThermoFisher Scientific, Waltham, MA, USA)], out of which the authors found 68 genes to be significantly upregulated and 30 to be significantly downregulated. GO enrichment led them to find that interferon-related pathways were enriched in significantly upregulated genes, which, based on the expression of interferon-stimulated genes (ISGs) in patients, led to the classification of k-means-clustered samples into ‘ISG high’ or ‘ISG low’ patients. Therefore, the authors concluded that there were two distinct molecular signatures found in COVID-19 postmortem lung tissue driven by ISGs.

In an attempt to classify the 31 patients infected with SARS-CoV-2 into similar ‘ISG high’ or ‘ISG low’ categories, the three GO terms mentioned by the authors (Nienhold et al., 2020) that were interferon-related were used to query for ISGs from the Gene Ontology knowledgebase (Gene Ontology Consortium, 2021). Sixty-six interferon-related genes were procured from the GO knowledgebase and then matched against the 398 genes provided (Nienhold et al., 2020). The resultant 14 genes were deemed as ISGs. From the DESeq resultant object for the whole-transcriptome data of our 41 samples, two subsets of the normalized matrix with 66 interferon GO term-related genes and 14 ISGs were made in order to visualize segregation (on the basis of ISGs) using PCA. For a majority of the samples (30/31, barring sample cov20), the variance remained unexplained along PC1 or PC2, on the basis of the 14 ISGs (Fig. S6). The 66 interferon-related genes obtained from GO were able to segregate the normal and COVID-19 samples much better, but were unable to illustrate the difference between the two groups of COVID-19 samples, which may have allowed us to label them as ‘ISG high’ or ‘ISG low’.

In order to further explore the presence of distinct categories/subtypes of patients among the COVID-19 group, the gene subset constraints to the DESeq resultant matrix were removed. GSVA was performed to quantitatively determine expression levels of respective gene sets based on DESeq normalized data. The GSVA scores for 14 ISGs and the 66 interferon-related GO genes did not show a significant difference (*P*<0.05) among the G1 and G2 COVID-19 sample groups and the normal (N) samples (box plots in Fig. S6).

To find the regulators orchestrating the massive rewiring of gene expression observed in COVID-19 patients, we investigated the enrichment of various targets of transcription factors (TFs) among our list of DEGs (Materials and Methods, Fig. S7A-C). Hepatocyte nuclear factor-4 α (*HNF4A*) and nuclear receptor subfamily 2 group F member 1 (*NR2F1*) were enriched in the upregulated DEGs in both groups (Fig. 3C). However, only *HNF4A* was overexpressed in the G2 patients when compared to the control group (Fig. 3D) as well as the G1 group (Fig. S3). These results corroborate the role of HNF-4α in COVID-19 and other chronic lung pathologies (Agudelo et al., 2020; Nardacci et al., 2021), potentially through its role as a master regulator of lipid metabolism, but only for the G2 group. This is further bolstered by lipid homeostasis-related GO terms being enriched in the upregulated genes of G2 when compared with G1. None of the other TF targets enriched in either upregulated genes or downregulated genes for both groups were themselves expressed in the same direction as their targets, indicating alternate regulation.

### Cell-type deconvolution analysis maps show an altered lung cellular profile

Gene expression rewiring can also be caused due to the change in the proportion of different cell types in the lung tissue of COVID-19 patients. To dissect this, first we performed GSVA using 57 gene expression signatures corresponding to different cell types in the lung tissue (Daamen et al., 2021). We found that 25 signatures (43.86%) showed a significant change between COVID-19 patients and control patients, most of them being downregulated in G2 patients (Fig. 4A). The only common signature between the G1 and G2 groups was the loss of alveolar macrophages, as reported previously (Liao et al., 2020). The G2 group showed an enrichment of signatures corresponding to erythrocytes, damage-associated M1 macrophages and neutrophils, suggesting acute inflammation and structural damage. The G2 group also showed loss of signature corresponding to cells in the lung parenchyma such as alveolar epithelial cells, endothelial cells and ciliated cells, alongside loss of platelet, suggesting prolonged lung damage and thrombocytopenia. Such reorganization of lung tissue was also accompanied by loss in signature corresponding to specific lymphocytes such as B-cells, activated T-cells, natural killer (NK)-T-cells and anergic T-cells, alongside loss of dendritic cells (DCs) and plasmacytoid DCs (pDCs), suggesting immune exhaustion.

To further dissect the finer details of lung physiology, we performed cell-type deconvolution to estimate the proportion of 58 different cell types using a published single-cell RNA-seq dataset from lungs (Travaglini et al., 2020) (Fig. S5B). In both the patient groups, we found a strong depletion in the proportion of IGSF21^+^ and EREG^+^ DCs, signaling alveolar epithelial cells and ‘Basophil Mast 2’ cells alongside enrichment in the proportion of lipofibroblasts, indicative of common pathophysiology. In the G1 group, we found a high proportion of proliferating NK-T-cells and ‘OLR1 Classical Monocytes’ alongside depletion of ‘Bronchial Vessel 2’ cells. Interestingly, two cell populations showed antagonistic changes in cell proportions namely proliferating basal and TREM2^+^ DCs. They were depleted in the G1 group but enriched in the G2 group of patients (Fig. 4B).

### *In silico* drug screening using connectivity maps

We performed connectivity map (CMap) analysis with differentially expressed genes from G1 versus normal and G2 versus normal samples to help predict the different set of drugs that could be used to reverse the molecular signature in the two types of patients. We identified 423,422 perturbagens and, among them, 136,460 were drug perturbagens that contain both therapeutic agents and inducers. We filtered them for compound data and A549 cell lines (adenocarcinomic human alveolar basal epithelial cells), and found 11,456 unique drug perturbagens for both groups. Among these, 1986 and 2048 perturbagens had negative connectivity scores for the G1 and G2 groups, respectively, implying their potential as therapeutic agents that could be screened further using *in vivo* experiments (Table S4) to identify potential drugs. Among the top therapeutic agents, we found curcumin, Ro-60-0175, talampicillin, FTI-276 and erastin as potential therapeutic agents for G1 patients, and SKF-96365, 2-chloroacetophenone, JNK-IN-5A, H-9 and cabazitaxel as potential therapeutic agents for G2 patients (Fig. 4C).

### Metatranscriptome analysis reveals difference in species richness and distribution between control and COVID-19 lungs

**Fig. 5.**
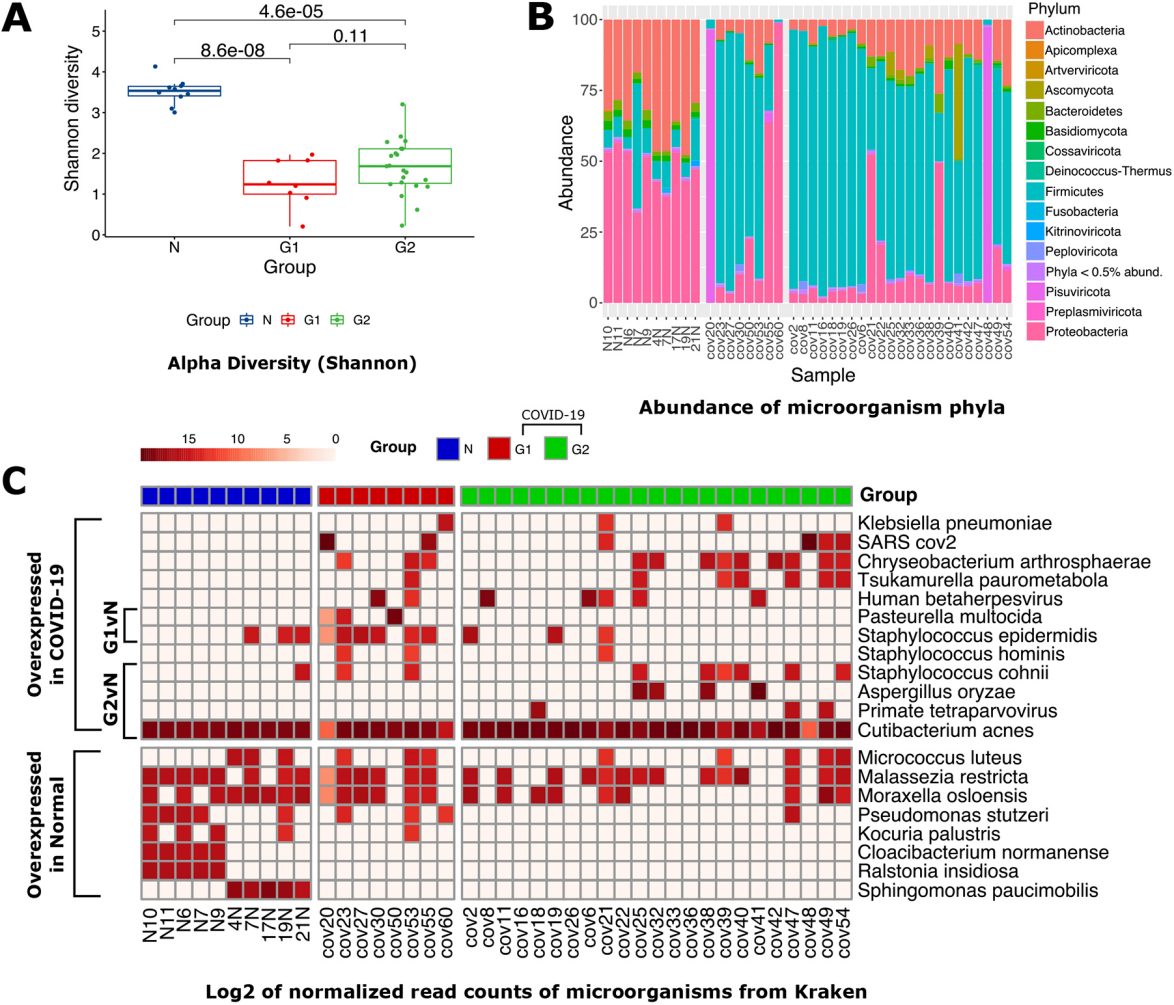
**Lung microbiome of COVID-19 and control patients.** (A) Box plots showing α-diversity (Shannon index) for N, G1, and G2 groups (*P*-values are based on Wilcoxon test). The box represents the 25-75th percentiles, and the median is indicated. The whiskers show the range. (B) Stacked bar plot representing the distribution of bacterial taxa at the phylum level. (C) Log_2_ of normalized read counts for the bacterial species present in at least three samples that were also present in both G1 and G2 as compared to the control group.

To identify the microbial signature in patient lung tissue, we performed a metatranscriptomic analysis (Fig. 1). Briefly, reads that did not align to the human genome (2.23±0.46%, indicated as mean±s.d.; Fig. S1C) were filtered for low complexity sequences (Fig. S1D) and bacterial rRNA, and then used for k-mer-based phylogenetic classification (Table S5). After taxonomic assignment, we found significant loss in species richness in both G1 (Wilcoxon test; *P*=8.6×10^−8^) and G2 (*P*=4.6×10^−5^) samples compared to control samples, with no significant difference between the two groups (*P*=1.1×10^−1^) (Fig. 5A). At the phylum level (Fig. 5B), Actinobacteria were found to be less abundant in both G1 and G2 samples compared to control samples (*P*=6.2×10^−4^ and *P*=1.5×10^−7^, respectively). We found depletion of Proteobacteria (*P*=4.2×10^−6^) and enrichment of Firmicutes (*P*=7.9×10^−6^) in G2 samples only (Fig. S8A).

To look at changes in the composition between COVID-19 patient groups at the species level and to reduce noise, we opted to include only those species with a minimum of 100 classified fragments (or reads) across at least three samples. We found 20 species distributed between the three groups of patients based on normalized counts per million (CPM) values. We found that some *Staphylococcus* species such as *S. cohnii* were enriched in both groups of COVID-19 patients, whereas other *Staphylococcus* species such as *Staphylococcus hominis*, *Staphylococcus epidermidis* and *Staphylococcus warneri*, along with the multidrug-resistant *Chryseobacterium arthrosphaerae*, were enriched in the G1 group. Additionally, the G1 group was also enriched for the pathogenic *P. multocida*, whereas the G2 group was enriched in other pathogens such as *Klebsiella pneumoniae* and *Tsukamurella paurometabola* (Fig. 5C).

### Analysis of the co-isolated SARS-CoV-2 genome and transcriptome

After phylogenetic classification of filtered microbial reads, only six out of the 31 (19.35%) COVID-19 samples were found to have more than 100 classified reads assigned to SARS-CoV-2 (Table S6). Three samples with greater than 10× depth and above 99% coverage were used for *de novo* genome assembly using SPAdes to obtain a single contig of length >29 kbp (corresponding to the full-length SARS-CoV-2 genome) from two samples, and 11 contigs spanning the entire genome were assembled from the third (deposited at GISAID with the accession IDs EPI_ISL_4392854, EPI_ISL_4392853 and EPI_ISL_4392851).

**Fig. 6.**
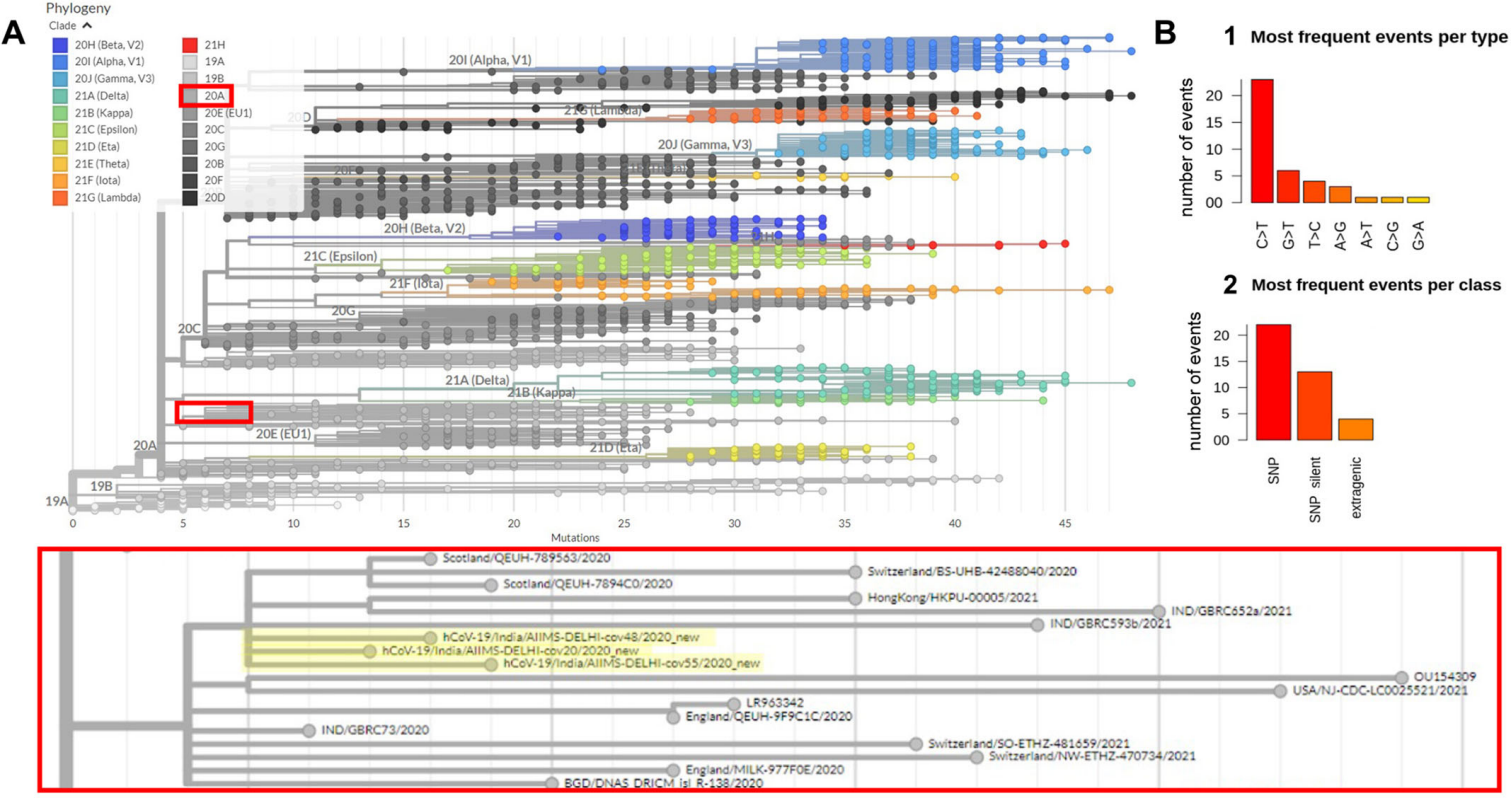
**Phylogenetic classification and analysis of recovered SARS-CoV-2 genomes.** (A) Phylogenetic analysis of the three high confidence SARS-CoV-2 genomes show that they all fall within the same nextstrain clade 20A and are next to each other in the phylogenetic tree. (B) Analysis of the mutations from all three genomes show that non-synonymous SNPs are the most common mutation type observed and that C>T base change was the most common mutation.

Consensus calling was used for variant calling by aligning to the Wuhan-Hu-1 reference strain (Materials and Methods). Phylogenetic analyses assigned these sequences to the GISAID clade GH or Nextstrain clade 20A in PANGO lineage B.1.36 (Fig. 6A) circulating in Europe, Asia and North America from September 2020 to March 2021 (Fig. S8C). Genomic sequence analysis revealed that cov20 had 12, cov48 had 13 and cov55 had 14 mutations with respect to the reference, with seven of these mutations in the spike protein region. Most of the mutations were single-nucleotide polymorphisms (SNPs; 56.41%), and C>T was the most frequently observed substitution (58.97%) (Fig. 6B; Table S6).

Given the depth of sequencing of SARS-CoV-2 virus in the three samples above, we were also able to estimate viral gene expression (Fig. S8B). We found that ORF10 had the highest expression level [mean transcripts per million (TPM) of 3.44 million], which was twice more than any other gene and is consistent with another report (Zhang et al., 2020). ORF7b had the lowest expression (mean TPM of 0.12 million), whereas the viral N gene, encoding the nucleocapsid and a common target for reverse transcription PCR (RT-PCR) diagnostic tests, had the second highest expression (mean TPM of 1.64 million). Since the control samples were collected much before the COVID-19 pandemic (Materials and Methods) and sequenced in the same batch as the COVID-19 samples, very few reads of COVID-19 appeared in our control samples, which were most probably a contamination during library preparation and were filtered out by setting a higher detection threshold for detecting COVID-19 in samples to at least 100 reads.

## DISCUSSION

Dissecting the molecular biology of primary lung tissue is essential for understanding drivers of mortality in severe COVID-19 infection to develop precision novel therapies and monitor disease prognosis. However, most large-scale cohort studies have been limited to non-invasive sampling from blood, nasopharyngeal fluid and bronchoalveolar lavage to delineate the effects of the disease in peripheral tissues (Wu et al., 2020b; Sanchez-Cerrillo et al., 2020; Xiong et al., 2020; Zhou et al., 2020), and consider all severe patients as a singular group. Most studies on lung tissue from severe cases of COVID-19 profiled either formalin-fixed, paraffin-embedded (FFPE) samples with compromised RNA or had a smaller sample size (about half the current study) (Wu et al., 2020b; Nienhold et al., 2020; Sanchez-Cerrillo et al., 2020; Xiong et al., 2020; Zhou et al., 2020). Here, we describe the molecular pathology of severe COVID-19 patients in the largest cohort lung tissue biopsies from 31 postmortem patients compared with biopsies of non-cancerous tissue from ten lung cancer patients as a control group. We found that COVID-19 patient lung tissue displayed two distinct molecular signatures defined by the lung transcriptome profiles. The lung transcriptome did not show indication of bacterial or fungal co-infections in severe COVID-19 patients.

The dominant ‘classical’ signature found in 74% of patients displayed a large-scale reorganization of gene expression characterized by loss in various parenchymal cells, the unfolded protein response, enhanced complement system supported by metabolic reprogramming, neutrophil upregulation and activated T-cell depletion. The rarer ‘cytokine release syndrome’ (CRS) signature found in 26% of patients showed minimal deviation in gene expression from the control group, but was marked by the proliferation of NK-T-cells, and enhanced production of cytokines such as IL1 and CCL19. A limitation of the comparisons drawn for significantly downregulated genes for COVID-19 would be that those genes are simply upregulated in cancer-adjacent normal tissues (due to them being upregulated in cancer) and is acknowledged as thus. TREM2^+^ DCs, which play a role in T-cell priming and are found in the BALF of severe COVID-19 patients (Liao et al., 2020), were enriched in the classical signature, but depleted in the CRS signature. Damage-associated M1 macrophage signature was enhanced in the dominant group and was depleted in the rare group, whereas the reverse pattern was seen for the protective M2 macrophages. Although a previous study by Nienhold et al. (2020) also described two distinct subtypes of COVID-19 based on the expression of ISGs, the two patient subtypes identified in this study could not be segregated based on ISG expression.

We propose two models to explain the disease signatures based on complement activation and failure to launch an adaptive immune response. The dominant signature represents ‘classic’ COVID-19 patients with an acute infection due to high initial viral load, as evidenced by the sustained unfolded protein response (Chan, 2014), leading to hyperinflammation through the complement system activating neutrophils. This hyperinflammation leads to lung damage (Fig. 2B) and recruitment of M1 macrophages. Also, these patients are unable to mount an adaptive immune response as evidenced by the depletion of T-cells, potentially due to direct viral infection or T-cell exhaustion due to alternate metabolic flux. The rarer signature might represent patients with cytokine release syndrome (Henderson et al., 2020; Tang et al., 2020; Hu et al., 2021) at low viral load, who are unable to deploy the complement system and depend on innate immunity through NK-T-cells. These patients might have characteristic ‘lung damage’, repaired by M2 macrophages. However, these patients too are unable to mount an adaptive immune response due to lymphopenia or diminished T-cell priming as a result of depleted TREM2^+^ DCs.

Although both groups of COVID-19 patients were given the same treatment, they displayed differences in the infection spectrum. Patients with the rare signature were specifically enriched for *S. epidermidis* and *P. multocida* despite administration of broad-spectrum antibiotics such as Azithromycin. The effect of broad-spectrum antibiotics can be perceived from the reduced species diversity in severe COVID-19 patients. Further, we found that the rare signature patients might have impaired immunity to deal with such infections through the downregulation of genes involved in chemokine receptor 6 (CCR6)-dependent bactericidal activity (including *DEFB1*), and the downregulation of genes involved in *de novo* biosynthesis of steroids from cholesterol (*HSD17B6*, *GREM2*, *FADS6* and *AADAC*). These characteristic changes in the lung microbiome warrant further investigation into the contribution of nasopharyngeal and airway microbiota in COVID-19 patients with lung complications.

It must be noted that the microbiome of lung tissue collected during aseptic surgery of cancer patients will significantly differ from the postmortem microbiome of COVID-19 patients. However, our results do highlight that there is a dysbiosis in lung tissue of patients with severe COVID-19, which is reinforced by the fact that lung tissues extracted in sterile conditions (normal samples) have more α-diversity than the COVID-19 postmortem lung tissues.

Despite most patients clearing the SARS-CoV-2 virus, we were able to recover the full genome from three patients that converged to the same consensus indicating clonal viral expansion. Upon analyzing the SARS-CoV-2 genome, we found that the ORF10 non-coding gene, with a low mutation rate and no selection pressure (Nguyen et al., 2021), had the highest gene expression with twice the number of transcripts as compared to the N gene, suggesting its potential use as a novel target for RT-PCR testing. Additionally, the three genomes recovered were from the same lineage (Fig. 6). With three genomes, one cannot significantly conclude whether these strains led to different trajectories in the two groups of patients, and if we had been able to retrieve complete genome sequences for more samples, we could interpret the cause in accordance with the strain of the virus.

In conclusion, our work supports further clinical investigation correlating prognosis by stratifying patients based on the circulating molecules involved in complement activation shown recently (Ma et al., 2021), or characteristic cytokines such as CCL19, implicated in COVID-19 mortality (Balnis et al., 2021). Such non-invasive stratification of patients can be used to test the efficacy of drugs identified in our study that reverse the molecular changes for the two patient signatures, such as cabazitaxel to treat ‘classical’ patients, or talampicillin to treat ‘CRS’ patients.

This is the first study analyzing the lung transcriptome in the Indian population, one of the worst affected countries in the world with over 524,000 COVID-19 mortalities. Although about one in seven people in the world come from India, data from Indian populations are often missing from such molecular investigations of diseases, and COVID-19 is no different. Our study bridges the gap of diversity in sampling various populations across the world that are affected by the pandemic. This is particularly of interest as in our recent work we demonstrate a significant contribution of genetics towards mortality in severe COVID-19 (Prakrithi et al., 2021). Therefore, we conclude by hypothesizing that the heterogeneity in the molecular signature of severe COVID-19 patients might be driven by patient genetics and can be used for candidate gene prioritization.

## MATERIALS AND METHODS

### Ethics statement

The study followed the standards and principles established by India's Directorate General of Health Services and Drug Controller General. Ethics approval was granted in writing from the Institute Ethics Committee of the All India Institute of Medical Sciences, New Delhi, India (IEC-538/05.06.2020, OP-28/05.02.2021). Consent was acquired from each patient's personal and/or family members for autopsy, biopsy and sample collection, in accordance with the Ethics approval.

### Patients and sample collection

This retrospective study examined 60 consecutive severe COVID-19 patients' autopsies performed at the All India Institute of Medical Sciences in New Delhi, India, between September 2020 and December 2020 for patients who spent the last few days of their lives in the intensive care unit (ICU). Minimally invasive postmortem tissue sampling was performed in less than 1 h on individuals with premortem PCR-confirmed SARS-CoV-2 infection at a biosafety level 3 postmortem facility. The control (uninfected) lung samples (*n*=10) represent healthy tissue taken from patients with lung cancer as part of standard medical evaluation during biopsy and/or surgical resection (>5 cm from site of tumor from early-stage non-small cell lung cancer patients undergoing curative surgical resection). These control samples were collected between August 2017 and July 2019. Paracancerous lung tissues have been taken as control for COVID-19 samples in other studies (Leng et al., 2020; Wang et al., 2021b). Lung tissue was harvested from the parenchyma region in accordance with a standard protocol for histology and snap frozen immediately for RNA extraction. Lung tissues were either fixed in 10% formalin, cut to the proper size and shape, and embedded in paraffin for histological examination, or treated with TRIzol (Life Technologies), snap frozen in liquid nitrogen, and kept at −80°C for RNA extraction. 31 out of 60 severe COVID-19 patient samples were chosen for analysis based on RNA yield and RNA quality (average RNA integrity number=6.09). ‘Severe’ COVID-19 was defined according to Ministry of Health and Family Welfare, Government of India guidelines (Updated Detailed Clinical Management Protocol for COVID19 adults dated 24 May 2021; available at https://www.mohfw.gov.in/pdf/UpdatedDetailedClinicalManagementProtocolforCOVID19adultsdated24052021.pdf). The guidelines define severe patients as the ones ‘characterized by a dysregulated immune response with hyperinflammation with subsequent development of acute respiratory distress syndrome (ARDS)’. These patients would be expected to have ‘acute respiratory infection with a history of fever or measured fever of ≥38°C, and cough, with onset within the last 10 days, and requires hospitalization’. According to the definition of ‘severe’ in the guidelines, the patient would present with severe pneumonia or ARDS or sepsis or septic shock. De-identified clinical information was extracted from patients’ medical records (Table S1).

### Histopathological evaluation

FFPE lung tissue blocks were processed and stained with Hematoxylin and Eosin (H&E) using a standardized procedure. Immunohistochemistry using the CD61 antibody (1:200, Clone EP65 RMab, Bio SB) was performed to visualize platelet components of microthrombi. Two thoracic pathologists (D.J. and A.N.) independently evaluated the slides. The following features were noted and documented: the extent of lung damage, injury, inflammation, presence or absence of hyaline membrane formation, lymphocyte infiltration, organizing pneumonia, alveolar fibrin deposition, fibrosis and histologic features of type 2 pneumocyte hyperplasia.

### RNA extraction, library preparation and sequencing

Total RNA was extracted from lung tissue using the Maxwell^®^ RSC Instrument and the Maxwell^®^ RSC Viral Total Nucleic Acid Purification Kit (Promega). The concentration of RNA and quality were measured with HS Total RNA 15nt methods (Agilent) or Qubit RNA HS Assay (ThermoFisher Scientific). The next-generation sequencing (NGS) library was prepared after cytoplasmic and mitochondrial rRNA depletion, using TruSeq Stranded Total RNA Gold kit as per the manufacturer's instructions (Illumina, 20020598). The libraries were then sequenced on an Illumina NovaSeq6000 platform with 2×150 base pair reads (details of statistics are given in Fig. S1).

### Host transcriptome analysis

Raw Illumina sequencing reads were checked for quality using FastQC (version 0.11.9) (https://www.bioinformatics.babraham.ac.uk/projects/fastqc/) followed by adapter clipping and trimming using Trimmomatic (version 0.39) (Bolger et al., 2014) with default parameters. Trimmed reads were then aligned to the human reference genome (GRCh38, GENCODE v36) (Schneider et al., 2017; Frankish et al., 2019) using STAR aligner (version 2.7.8a) (Dobin et al., 2013). FeatureCounts (subread package version 2.0.1) (Liao et al., 2014) was used to quantify gene expression. Quality checks were performed at each step using the MultiQC tool (version 1.10.1) (Ewels et al., 2016). Differential gene expression analysis was performed using the DESseq2 package (version 1.30.0) (Love et al., 2014) in R (version 4.0.3). The analysis was performed by removing the effects of confounding variables such as age and gender (Table S1) using the appropriate design formula. Genes with Benjamini–Hochberg-adjusted *P*-value <0.05 and absolute log_2_ fold change >1 in either direction were taken as significantly differentially expressed and shrunken log_2_ fold change values were used for further analysis. ClusterProfiler package (version 3.18.0) (Yu et al., 2012) was used for the GO term Over Representation Analysis (ORA) of differentially expressed genes. GSVA package (version 1.38.2) (Hänzelmann et al., 2013) was used for all GSVA analysis and heatmaps of GSVA enrichment scores were visualized using the package pheatmap (version 1.0.12) (https://cran.r-project.org/web/packages/pheatmap/index.html). Enriched gene sets had a corresponding change in GSVA enrichment scores with *P*<0.05 using Wilcoxon test between the two groups compared. Box plots and other visualizations were made using the ggplot2 package (version 3.3.3) (Wickham, 2011). All statistical tests were performed using functions from the base or stats package in R. For identification of transcription factors driving gene expression, we used the Enrichr tool (Kuleshov et al., 2016), using lists of genes upregulated in severe COVID-19 samples (and sub-groups as identified in the paper) when compared with controls.

### Curation of gene lists

Gene lists for GSVA were manually curated from various sources (Table S2). GSVA plots of cell types are based on published gene lists (Daamen et al., 2021). Gene lists for fibrosis and extracellular matrix (Extracellular Structure Organization) were taken from Wu et al. (2020b). The surfactant protein gene list was referenced from Islam and Khan (2020). Gene lists for interferons, chemokines, interleukins, their receptors and other innate immune related pathways were sourced from HGNC (Vasiliou et al., 2021), ImmPort (Bhattacharya et al., 2018) and published gene lists (Daamen et al., 2021). KEGG pathways were utilized from MsigDB (Liberzon et al., 2011). The gene list for host proteins that interact with SARS-CoV-2 was referenced from Gordon et al. (2020).

### Cell deconvolution analysis

Multi-subject single cell deconvolution (MuSic_0.2.0) (Wang et al., 2019) was used to predict the relative composition of different cell types from bulk RNA-seq samples using existing single-cell RNA-seq (scRNA-seq) dataset as reference. From the relative composition of cell types, ‘Hedges’ g effect size’ was measured using effsize_0.8.1 R package (Torchiano, 2016). Changes in cellular proportions comparing G1 versus N and G2 versus N were plotted for only those cell types that gave finite values in both G1 and G2, using a ‘scatterplot’ using the ggplot2 (Wickham, 2011) R package.

### CMap analysis

CMap (Lamb et al., 2006) analysis was performed using the online portal https://clue.io/cmap to determine perturbagens (potential drugs reversing the aberrant gene expression) using the L1 version of CMap with L1000 data repository, and Touchstone dataset as a benchmark for assessing connectivity among perturbagens and individual query option. The pertubagens were further filtered for potential therapeutic drugs.

### Metatranscriptomic analysis

Reads not mapped to the human genome were filtered to remove low complexity (entropy≥0.7) human rRNA and mitochondrial reads using BBMap toolkit (version 38.90) (Bushnell, 2014). The filtered unmapped reads were then input into Seal (from the suite of bbtools) and binned into bacterial rRNA (using SILVA bacterial rRNA database) (Quast et al., 2013), human genome (GRCh38) and microbial bin. Taxonomic classification of reads was carried out on the microbial bin using Kraken2 (Wood et al., 2019), using organisms with at least 100 reads at the genus level for classification and confidence level of 0.3. The α-diversity (Shannon diversity index) and bacterial taxon abundance was assessed using the PhyloSeq package (version 1.34.0) (McMurdie and Holmes, 2013).

### SARS-CoV-2 genomic and transcriptomic analysis

All COVID-19 samples with detectable SARS-CoV-2 reads were taken for further analysis. Filtered microbial reads from these samples were aligned against the SARS-CoV-2 reference genome (Wu et al., 2020a) using BBMap (version 38.9) (Bushnell, 2014). Depth and coverage of the viral genome were obtained using samtools (version 1.9) (Li et al., 2009). Full-length genomes were assembled for samples with high depth and coverage using SPAdes. The SARS-CoV-2 genomes were classified using GISAID (https://www.gisaid.org/), PANGO database (https://cov-lineages.org/) and nextclade (https://clades.nextstrain.org/) and were placed in a phylogenetic tree created using nextstrain (https://nextstrain.org/). Information on mutation types and frequency was obtained from http://giorgilab.unibo.it/coronannotator/. Additional information on strain B.1.36 was obtained from https://outbreak.info/situation-reports?pango=B.1.36. Transcriptome analysis was performed by aligning filtered viral reads to the reference strain (Wuhan-Hu-1) using Bowtie2 (Langmead and Salzberg, 2012). Read counts for the viral genes were obtained using featureCounts and normalized to TPM values.

## Supplementary Material

Supplementary information
